# Evaluation of the Current Perspectives on Letters of Recommendation for Residency Applicants among Plastic Surgery Program Directors

**DOI:** 10.1155/2012/728981

**Published:** 2012-04-02

**Authors:** K. Shultz, R. C. Mahabir, J. Song, C. N. Verheyden

**Affiliations:** ^1^Division of Plastic Surgery, Scott & White Healthcare and Texas A&M Health Science Center, Temple, TX 76508, USA; ^2^Department of Biostatistics, Scott & White Healthcare, Temple, TX 76508, USA

## Abstract

*Background*. The goals of this project were to evaluate the current perspective on letters of recommendation and to assess the need for, and acceptance of, a more standardized letter of recommendation (LOR). *Methods*. An eight-question survey was distributed to plastic surgery program directors. A five-point Likert scale was selected as a means of quantifying the participants' responses to the survey. *Results*. Twenty-eight of 71 program directors (39.4%) completed the survey. The majority of participants felt that current LOR did not offer a realistic way to compare applicants (mean ± SD, 2.9 ± 0.8). While most agreed that increasing the objectivity of LOR would be valuable in comparing applicants (mean ± SD, 4.1 ± 0.9), the overall average response to whether a more standardized letter format would improve the resident selection process remained only slightly better than neutral (mean ± SD, 3.5 ± 1.2). Most of the chairmen supported the notion that familiarity with the author of the LOR strengthened the recommendation (mean ± SD, 4.5 ± 0.6). *Conclusion*. The majority of plastic surgery program directors would like more objectivity in comparing applicants but are ambivalent about a standardized letter of recommendation.

## 1. Introduction

Plastic surgery residency candidates are increasingly competitive which, in turn, raises the complexity of selecting the most qualified applicants. Rating applicants is a multifactorial process involving the assessment of many variables including grades, class rank, performances at subinternship rotations, USMLE scores, AOA and other honors, research accomplishments, and letters of recommendation.

In a recent study by Janis and Hatef, a survey was conducted to assess the current method of plastic surgery resident selection and program directors' satisfaction with the process. The study demonstrated that although many directors were displeased with the current system, few changes have been made towards improvement. Interestingly, letters of recommendation were found to be a valuable criterion for selection, yet letters of recommendation remain primarily a subjective measure of a candidate's character and quality. Attrition rates from residency programs were deemed unacceptably high, which may reflect a problem with applicant selection. It was concluded that increasing objectivity might raise overall satisfaction [1].

Recently, letters of recommendation have been under scrutiny across many specialties. While some feel that letters of recommendation are an invaluable component in the overall evaluation of each applicant, others argue that, as the number and quality of applicants rise, current letters fail to distinguish excellent applicants from the average [2–6]. Studies seem to suggest that very few provide comparative data that allow for any objective analysis of candidates [2, 4, 7, 8]. A study by O'Halloran et al. highlighted some of the deficiencies in letters of recommendation in radiology, showing that when presented identical letters, two experienced reviewers were rarely in agreement regarding candidate quality. The study also showed that traditional letters of recommendation were often missing key information deemed necessary to adequately assess residency candidates [6].

The ambiguity and seeming lack of specificity of the current narrative LOR led to the development of a standardized letter of recommendation (SLOR) now used by the specialty of emergency medicine [4, 9]. Emergency medicine program directors have described a very positive experience with the new SLOR and as a result other medical specialties are considering ways to standardize their LOR [5]. To that end, the American Council of Academic Plastic Surgeons (ACAPS) recently created an SLOR in an attempt to provide a more objective evaluation of residency candidates. It is available online on the ACAPS website (http://acaplasticsurgeons.org/multimedia/files/Letter-of-Recommendation-Form.doc; see Supplementary Material available online at doi: 10.1155/2012/728981). Although other studies have looked at the overall candidate evaluation and resident selection process, no study has focused on LOR in plastic surgery. The goals of this project were to evaluate the current perspective on letters of recommendation among plastic surgery program directors, to measure the utility and ease with which directors are able to extract information and draw accurate conclusions about the various residency candidates, and to assess the need for, and acceptance of, a more standardized letter of recommendation. 

## 2. Materials and Methods

An eight-question survey was created to assess the current perception of LOR in the plastic surgery resident selection process. The questions focused on either the utility of LOR in the resident selection process or the program directors' assessments of the benefits of a more standardized letter format. The questionnaire was distributed and collected at the ACAPS annual meeting prior to the presentation of the SLOR prototype. Each member was asked to complete the survey upon entering the meeting.

A five-point Likert scale was selected as a means of quantifying the participants' responses to the survey. A “5” meant that they strongly agreed, while “1” was strongly disagree. A rating of “3” represented a neutral stance, “2” meaning somewhat disagree, and “4” for somewhat agree. At the end of the survey, an area was allotted for comments.

Refer to (Supplementary Material) for the list of eight ranked survey statements.

Response scales of each of eight questions were summarized using the mean plus or minus the standard deviation for the entire group and according to the type of program. Integrated and independent programs were compared in each of eight questions utilizing the Wilcoxon rank-sum test. Q1, Q5, and Q8 were selected for further analysis in an attempt to compare perceived quality of LOR with desire for an SLOR. Response scales of three or less were merged, and response scales of four or five were merged for questions Q1, Q5, and Q8. The homogeneity of the marginal distributions of Q1 and Q5 with Q8 were assessed using McNemar's test. Association of Q1 and Q5 with Q8 was assessed utilizing Fisher's exact test. *P* value of less than 0.05 indicated a statistical significance. SAS version 9.2 (SAS Institute Inc., Cary, NC) was used for data analysis.

## 3. Results

**Figure 1:**
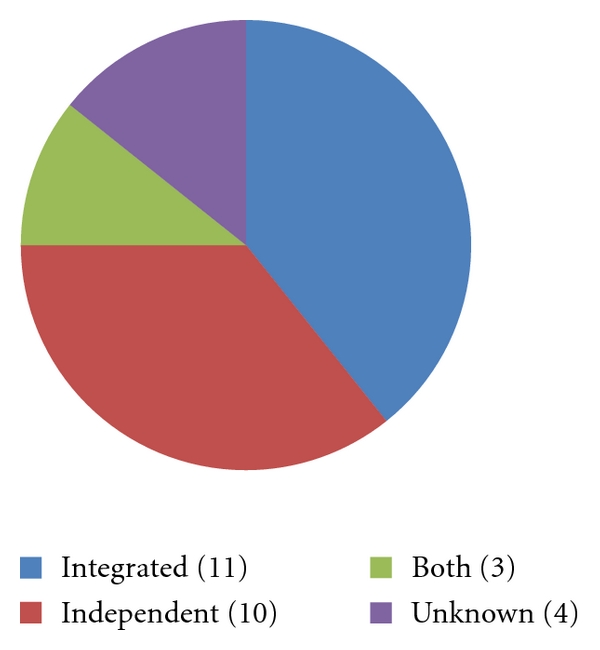
Breakdown of 28 respondents by program type. This Figure represents the breakdown by program type. The four “unknown” programs represent the four survey respondents who did not identify their program as a specific type (integrated, independent, or both).

 A total of 28 of all 71 program directors nationwide (39.4%) completed the survey. Not all 71 program directors were in attendance at the ACAPS meetings at the time of the survey.  Figure 1 depicts the breakdown of respondents by program type (integrated, independent, both, or unknown).  Table 1 shows what parties are involved in reviewing letters of recommendation according to the 28 program directors that responded to the survey. 

Table 1 shows who is responsible for reviewing letters of recommendation among those programs that responded to the survey. Many programs employ multiple parties to review letters.

The majority of program directors felt that current LOR failed to definitively offer a realistic way to compare applicants (2.9 ± 0.8). While most agreed that increasing the objectivity of LOR would be valuable in comparing applicants (4.1 ± 0.9), the overall average response to whether a more standardized letter format would improve the resident selection process remained only slightly better than neutral (3.5 ± 1.2). However, upon further stratifying the responses, there was a significant difference in the proportion of those that strongly or somewhat agreed with statement five versus statement eight. Thus, more participants strongly or somewhat agreed that increasing the objectivity of LOR would be valuable than that a standardized letter format would improve the overall selection process. However, no significant association was detected between responses to Q5 and responses to Q8 (Table 2).

Table 2 summarizes the association between Q1, Q5, and Q8. No significant difference was detected in the marginal distributions of Q1 and Q8. A significant difference was detected in the marginal distributions of Q5 and Q8. The percentage of 4 and 5 ratings for Q5 was significantly higher than that of Q8. Participants agreed more with Q5 than Q8.

Most of the program directors supported the notion that familiarity with the author of the LOR strengthened the recommendation (4.5 ± 0.6). Increasing the number of letters of recommendation did not allow for a more accurate assessment of the applicant (2.8 ± 1.0). Most respondents remained neutral in their stance on the LOR as a predictor of resident performance (3.1 ± 0.9). When further stratifying the results based on ratings for statements one and eight, there was no significant difference in the proportion of those who strongly or somewhat agreed that LOR were valuable predictors of resident performance compared to those who strongly or somewhat agreed that SLOR would enhance the overall resident selection process.

Table 3 presents the results for all respondents, and Table 4 stratifies responses by program type. When comparing survey results across program formats, no significant differences were apparent (Table 4).

Table 3 summarizes the response scales of all 28 subjects. One subject marked 2.5, and one subject circled both 2 and 3 for question 1. We performed 2 separate analyses considering those cases as 2 and 3. Q4 and Q5 had the highest mean response scale (4.5 and 4.1, resp.), and Q2 and Q3 had the lowest mean response scale (2.9 and 2.8, resp.). Questions Q4–Q8 had a median of 4 or 5, while questions Q1–Q3 had a median of 3.

Table 4 summarizes response scales according to the type of program. Integrated and independent programs were compared in the response scale of each question. No significant differences were detected between integrated and independent programs in each of the 8 questions (all *P* values were greater than 0.05).

## 4. Discussion

Survey results recently published by the National Resident Matching Program illustrated the importance of letters of recommendation in the plastic surgery resident selection process [10]. Seventy-nine percent of programs completing the survey indicated LOR from plastic surgeons were utilized in choosing applicants to interview, the highest percentage of any other factor cited. LOR by authors within the plastic surgery field were deemed very valuable, receiving an importance rating of 4.7 out of 5 [10]. This seems to support our finding that LOR written by familiar authors, likely those within the plastic surgery field, strengthen the recommendation and perhaps increase the predictive value in selecting qualified applicants. Traditional LOR in general, however, were deemed to be of only marginal value, reiterating the importance of the author in providing validity and perceived objectivity.

Morgenstern et al. looked at ways to improve the LOR, noting that “superlative inflation” has become the norm in letter writing. Many authors find it necessary to use extreme diction in order to avoid potential disservice to students by providing insufficient praise [5]. Such semantics often make interpretation difficult. Although most respondents of our survey somewhat or strongly agreed that increasing the objectivity of LOR would be valuable in comparing candidates, a portion of program directors felt the opposite. Perhaps interpreting letters is a skill that is acquired, and one must learn to decipher the “language of the letters.” Thus, those who are better skilled at decoding, view letters as being more objective and perceive less need for standardization.

In 1995 the specialty of emergency medicine adopted an SLOR model in attempt to curtail “adjective inflation” and increase accurate information about each applicant. The standardized format has been positively received by emergency medicine program directors and is now commonly used in evaluating prospective residents. A survey of the program directors after its implementation highlighted a few of the perceived values of the SLOR. Results of the poll suggested that it was easier than traditional LOR to read and incorporate into a ranking scheme. Readers of the SLOR were better able to discriminate differences between candidates, and evaluators found it easier to complete. Other studies suggest that the emergency medicine SLOR has resulted in better interrater reliability and requires less time to interpret [4].

The present study indicates that, while a majority desire more objectivity, most plastic surgery program directors remain neutral in their perception of the need for a standardized letter of recommendation. Letters of recommendation by authors familiar to the reader and from within the plastic surgery field remain a valuable part of applicant evaluation. A more standardized letter format may serve to supplement the narrative letters of recommendation currently used, particularly when an unfamiliar author outside of the plastic surgery field writes the letter.

Two major training models exist for plastic surgery, independent and integrated. In the integrated model, residents are accepted directly from medical school to begin their six-year training which consists of a primarily general surgery focus and transitions into plastic surgery exclusively the final few years. The independent model requires prerequisite training prior to joining the program. The resident must first complete either 36 months of general surgery training or complete a formal training in general surgery or a number of other surgical subspecialties. Surprisingly no statistically significant difference was detected between responses from program directors of the two different program types. It was hypothesized that program directors of independent programs would find greater value in current LOR in predicting resident performance, given the far greater surgical resident experience for the letter writer to critique. However, the responses suggest that neither subset of program directors, on the average, felt that LOR were good predictors of resident performance or offered a realistic way to compare applicants.

The survey used was completed prior to the introduction of the SLOR, thus the respondents' opinions represent those prior to the approval of the proposed change. It is unknown whether responses would have changed if the survey was conducted after the standardized letter model was available to program directors. An additional limitation to the present study is the low response with approximately 28 completed surveys of the 71 program directors nationwide. Although most program directors indicated that they were involved in reviewing LOR, the chair of the division/department for 21 of the 28 programs also reviewed LOR. The current study did not survey the opinions of the chairs; their perspectives may prove valuable and should be studied in the future. Further studies are necessary to assess changes in perspectives as the field of plastic surgery evolves, competition for residency positions increases, and program directors become more familiar with the standardized letter prototype. Potentially useful long-term follow-up studies include looking at the number of authors/programs that use the SLOR format and opinions and experiences regarding its use. Long-term prospective studies comparing traditional LOR to SLOR for individual plastic surgery applicants should be conducted to assess predictive value of resident successful completion of residency.

## 5. Conclusion

Despite the subjective nature of LOR, they remain a vital component of the applicant evaluation. Although plastic surgery program directors desire more objective data on which to base their resident selection, many remain ambivalent to moving to a standardized format. The recently proposed SLOR should be considered as a supplement to current LOR, particularly when unfamiliar authors outside the field of plastic surgery write letters.

## Supplementary Material

The letter of recommendation template was created by ACAPS in an effort to help residency programs compare across candidates. There are both objective and subjective sections as many authors desired a section where they could still add subjective content.

## Figures and Tables

**Table 1:** Party responsible for reviewing letters of recommendation.

Party responsible for reviewing letters of recommendation	Total number of programs (out of the 28 respondents)	Percentage of programs (out of the 28 respondents)
Residency program director	25	89.3%
Chair of the division/department	21	75.0%
Residency coordinator	6	21.4%
Resident selection committee	13	46.4%
Other	6	21.4%

**Table 2:** Association between questions.

		Q8. A standardized letter of recommendation would improve the overall selection process.		*P* value*	*P* value**
		Scales 1–3	Scales 4-5		
Q1. The current letters of recommendation are valuable predictors of resident performance.	Scales 1–3	8	10	0.1796	1.0000
	Scales 4-5	4	6		

Q5. Increasing the objective nature of the letters of recommendation would be valuable in comparing candidates.	Scales 1–3	4	1	0.0391	0.1331
	Scales 4-5	8	15		

*McNemar's test was used.

**Fisher's exact test was used.

**Table 3:** Subject characteristics (entire group).

Question	Mean	SD	Median	Minimum	Maximum
PQ1*	3.1	0.9	3.0	2.0	5.0
Q1**	3.2	0.8	3.0	2.0	5.0
Q2	2.9	0.8	3.0	1.0	4.0
Q3	2.8	1.0	3.0	1.0	4.0
Q4	4.5	0.6	5.0	3.0	5.0
Q5	4.1	0.9	4.0	1.0	5.0
Q6	3.9	0.9	4.0	1.0	5.0
Q7	3.6	1.0	4.0	1.0	5.0
Q8	3.5	1.2	4.0	1.0	5.0

*2.5 was considered as 2.

**2.5 were considered as 3.

**Table 4:** Subject characteristics according to type of program (mean ± SD are listed).

Question	Integrated (*N* = 11)	Independent (*N* = 10)	Both (*N* = 3)	None (*N* = 4)	*P* value***
Q1*	3.0 ± 1.0	3.0 ± 0.5	4.3 ± 0.6	2.8 ± 1.0	1.0000
Q1**	3.0 ± 1.0	3.0 ± 0.5	4.3 ± 0.6	3.3 ± 0.5	1.0000
Q2	2.6 ± 0.9	3.1 ± 0.7	3.7 ± 0.6	2.3 ± 0.5	0.2463
Q3	2.7 ± 1.1	2.6 ± 0.8	4.0 ± 0.0	2.5 ± 1.0	0.7105
Q4	4.6 ± 0.5	4.4 ± 0.7	4.7 ± 0.6	4.3 ± 0.5	0.4672
Q5	4.0 ± 0.8	3.9 ± 1.1	4.3 ± 1.2	4.5 ± 0.6	0.8748
Q6	4.1 ± 0.8	3.7 ± 1.1	4.0 ± 0.0	4.0 ± 0.8	0.2921
Q7	3.7 ± 1.3	3.3 ± 0.8	4.3 ± 0.6	3.3 ± 0.5	0.2158
Q8	3.3 ± 1.1	3.3 ± 1.5	4.0 ± 1.0	4.0 ± 0.8	0.7698

*2.5 was considered as 2.

**2.5 were considered as 3.

***P value to compare integrated and independent programs.
