# Supplementary material for: Evaluation of the Current Perspectives on Letters of Recommendation for Residency Applicants among Plastic Surgery Program Directors
Source: Plast Surg Int. 2012 Apr 2;2012:728981. doi: 10.1155/2012/728981 (PMC3335712; doi:10.1155/2012/728981)
Supplement: Supplementary file 1 — The letter of recommendation template was created by ACAPS in an effort to help residency programs compare across candidates. There are both objective and subjective sections as many authors desired a section where they could still add subjective content. [file 728981.f1.pdf]

2011-2012 APPLICATION SEASON

Plastic Surgery Residency Recommendation Form

Applicant's Name: AAMC ERAS ID No.

Reference Provided By:

Present Position: Email:

Institution: Telephone Number:

A. Background Information

1. How long have you known the applicant?
2. Nature of contact with applicant: (Check all that apply)
   - Know indirectly through others/evaluations or occasional contact
   - Direct observation- plastic surgery rotation
   - Direct observation- research
   - Advisor

3. If this candidate rotated in plastic surgery, what grade was given?

Honors High Pass Pass Low Pass Fail

Optional: One Key Comment from Faculty or Residents' Eval:

Please circle the most appropriate response:

**ACADEMIC POTENTIAL**	**SURE THING; HAS A SLOT**	**HIGH**	**LEANING TOWARD**	**NOT SURE**
**COMPARED TO OTHER RESIDENTS**	**THE BEST**	**TOP 5%**	**TOP 20%**	**AVERAGE**
**WORK ETHIC**	**SUPERIOR**	**EXCELLENT**	**VERY GOOD**	**AVERAGE**
**INITIATIVE**	**HARD TO CONTAIN**	**SELF STARTER**	**TEMPERED**	**NEEDS GUIDANCE**
**TECHNICAL ABILITIES**	**A WHIZ**	**EXCELLENT**	**ABOVE AVERAGE**	**AVERAGE**
**AFFABILITY**	**UNEQUALED**	**VERY GOOD**	**ABOVE AVERAGE**	**OK**
**HONESTY**	**UNEQUALED**	**EXCELLENT**	**AVERAGE**	**NOT ENOUGH EXPOSURE TO TELL**

☐ Please call me about this applicant.

What is this applicant's strongest point?

We <u>all</u> have faults and flaws. What is the applicant's weakest point(s)?

If you are (OR WERE) associated with a plastic surgery residency that has a position available for the match in which this candidate is applying, please estimate where this candidate would rank on your list:

1-3　　4-7　　8-10　　greater than 11

Written Comments

Signature: ______________________　　Dated: ______________________

☐ **STUDENT HAS WAIVED RIGHT TO SEE THIS LETTER**
