# Supplementary material for: Evaluation of the Current Perspectives on Letters of Recommendation for Residency Applicants among Plastic Surgery Program Directors
Source: Plast Surg Int. 2012 Apr 2;2012:728981. doi: 10.1155/2012/728981 (PMC3335712; doi:10.1155/2012/728981)
Supplement: Supplementary file 2 [file 728981.f2.pdf]

Appendix B

The eight ranked survey statements were:

Q1. The current letters of recommendation are valuable predictors of resident performance.

Q2. Letters of recommendation offer a realistic way to compare applicants.

Q3. More letters of recommendation allow for a more accurate assessment of the applicant.

Q4. Familiarity with the author of the letter strengthens the recommendation.

Q5. Increasing the objective nature of the letters of recommendation would be valuable in comparing candidates.

Q6. Non-cognitive (qualitative) measures are important in ranking applicants.

Q7. Letters of recommendation are valuable resources in assessing an applicant’s non-cognitive attributes.

Q8. A standardized letter of recommendation would improve the overall selection process.
